# The Baby: Its Manners and Its Morals

**Published:** 1893-09-09

**Authors:** 


### A PHYSIOLOGICAL LANTERN.

### II.—THE BABY: ITS MANNERS AND ITS MORALS.

It is said that John Wesley used to advise that babies might be corrected by whipping so soon as they had reached the age of four weeks. If Wesley did give such advice, which we do not believe, it is certain, not only that he was no physiologist, but that, so far as babies were concerned, he was no philosopher. Babies have no morals, and cannot have any. The Calvinistic idea that they are "born in sin" is preposterous. Its crudity is worthy of the darkest of unscientific ages. The Bible, which is one of the soberest as well as one of the most venerable of books, lends no authoritative countenance to any such notion. A Hebrew poet, in a moment of intolerable remorse, experienced a consciousness of personal depravity so profound that nothing he felt could measure the case but a confession of absolute, life-long, ingrained and congenital baseness. But to found upon a single poetical exaggeration a doctrine that every human infant is born in sin and deserves eternal condemnation, is one of those aberrations of the human mind which sober and scientific thinkers feel it their duty to endeavour to correct at any cost.

Regarded soberly and sympathetically, without prejudice on the one hand, or stolid mental dulness on the other, a newborn baby is a wonder and a beautiful miracle. Its form and figure are such that painters in all ages have delighted to paint the child. The physiologist sees a thousand things in the baby which the painter never saw. Where was this wonderful creature twelve months ago? How did it acquire this perfect outward form, those beautiful and finished internal structures and functions, in this brief period of time? Its eye is a world of wonders in itself, its ear another, its speaking and breathing organs another.

The greatest miracle of all is the power of expansion, physical, mental, moral, which lies encased in that small form. What will the child be thirty years hence? A Shakespeare perhaps. Perhaps a Samson Agonistes.

The physiologist sees in the baby a physical form endowed with life, having physical necessities and physical possibilities. He recognises the sacred duty of bringing that physical form to such physical perfection as it is capable of. Surely herein the physiologist is a priest. This organism is to a certain extent committed to him by destiny or providence, and committed to him because he has knowledge and experienced capacity to render to the baby exactly those services which nature has left it in need of. The possession of the capacity points out the physiologist as the natural and ordained priest of the child's body. The physiologist is the appointed modern protector of humanity in its crude, early, and incapable stages.

A baby, though incapable of morals in the theological sense, is not incapable of discipline. On the contrary, one of the first essentials for happy and prosperous babyhood is discipline. But the little creature cannot practice self-discipline. The discipline must come from without. And it is obvious that the discipline is not to be imparted by word of mouth, or by expressed law and statute, or by punishment for wrong doing or reward for right doing. What then is the natural instrument of discipline for the baby in the first months of its life? Habit, clearly; and nothing but habit. Merely by regularity of habit, regular feeding, regular washing and dressing, regular hours of sleep, regular periods of waking, regular attention to all physiological wants, the six months' old baby becomes one of the most disciplined creatures alive. And this disciple carries with it happiness and health in their fullest measure. The physiologist who secures this for the infants committed to his charge is a most successful bishop and priest of the bodies of his baby flock.

Not only the first few months, but the first few years of the baby's life, belong almost exclusively to the domain of the physiologist. Food, clothing, and lodging are the primary things for a child. Next to these the chief thing is to provide it, not with amusements, but with the means of amusing itself. But is the child to be taught no manners—no morals? Certainly; but chiefly by one method of instruction. In its earlier babyhood it was taught by regularity of habits; now, at the age of two, three, four, up to ten or twelve, it is to be taught almost exclusively by suitable object lessons. Models of conduct, models of character are to be set before it for its conscious, but more for its unconscious study and imitation. The parents, the older children, the servants are the child's natural object lessons. It is to them that the law must be laid down; it is upon them that punishment should be inflicted, and to them that rewards must be promised. The mother who possesses a whip or a rod should in most cases use it upon herself. During the period of childhood from birth to the seventh or eighth year of age life should be easy, careless, joyous, and totally unconscious of strain.

When should a child begin to learn religion? It should begin as soon as it has learnt to recognise the human creatures that are about it. But the religion it should then learn is the religion it sees in the faces of its parents and nurses, that it hears in the tones of their voice, that it feels in the touch of their hands. The natural divinities of the child are its parents; its natural catechism their conduct; its natural hope their approval; its natural fear their frown.

The idea of "sin," which many religious teachers associate with early childhood is, from a physiological point of view, absurd. A young child cannot sin. No one who cannot fully comprehend law can fully comprehend the infraction of it; and no such person can "sin." Can the child fully comprehend law and its infraction? The young child is no more capable of sin than is the horse or the sheep. The idea of a "sinful" horse, or a "sinful" sheep, is too ludicrous. The child may be wilful, passionate, inconsequent. But if he is, it is either because he has inherited these peculiarities from his parents, or because he has not been trained by reasonable and kindly trainers. But will anybody blame a child for the peculiarities he has inherited from his parents, or for the habits he has acquired as the result of incompetent training? The child, like the man or the woman, can only be blamed for that which he does himself by calm choice and of deliberate purpose. But when does the child calmly choose? When does he deliberately purpose?

On this question of the innocence of childhood physiology is entirely at one with the New Testament. The founder of Christianity himself, by a deliberate act, pointed out the young child as the model and object lesson, for men, of the kingdom of the heavens. The spoilt child is like the spoilt piece of porcelain, spoilt by someone else's handling, not by his own choice or will. The normal child, normally brought up, leaves nothing to be desired in the way of physical beauty, or of mental sweetness and simplicity. If all human minds were as sweet and wholesome as his, we need ask no more. The stern pharisee, the narrow-minded bigot, the man who "wastes no time in thinking," will oppose this, and will insist upon the "depravity," the "sin," and the "punishment" as before. He who does these things must be prepared to take the consequences. Physiology denounces as stern a "woe" as the New Testament upon all these, whether preachers or parents, that "offend the little ones," who are the sweetest, truest, and most natural human types of the beautiful kingdom of the invisible.

